# Assembly of Spinach Chloroplast ATP Synthase Rotor Ring Protein-Lipid Complex

**DOI:** 10.3389/fmolb.2019.00135

**Published:** 2019-11-29

**Authors:** Olga Novitskaia, Pavel Buslaev, Ivan Gushchin

**Affiliations:** ^1^Research Center for Molecular Mechanisms of Aging and Age-Related Diseases, Moscow Institute of Physics and Technology, Dolgoprudny, Russia; ^2^Nanoscience Center, Department of Chemistry, University of Jyväskylä, Jyväskylä, Finland

**Keywords:** membrane protein, membrane insertion, complex assembly, annular lipids, protein-lipid interactions

## Abstract

Rotor ATPases are large multisubunit membrane protein complexes found in all kingdoms of life. The membrane parts of these ATPases include a ring-like assembly, so-called c-ring, consisting of several subunits c, plugged by a patch of phospholipids. In this report, we use a nature-inspired approach to model the assembly of the spinach (*Spinacia oleracea*) c_14_ ring protein-lipid complex, where partially assembled oligomers are pulled toward each other using a biasing potential. The resulting assemblies contain 23 to 26 encapsulated plug lipids, general position of which corresponds well to experimental maps. However, best fit to experimental data is achieved with 15 to 17 lipids inside the c-ring. In all of the simulations, the lipids from one leaflet (loop side of the c subunit) are ordered and static, whereas the lipids from the other leaflet are disordered and dynamic. Spontaneous permeation of water molecules toward Glu61 at the active site is also observed. The presented assembly approach is expected to be generalizable to other protein complexes with encapsulated lipid patches.

## Introduction

**Figure 1:**
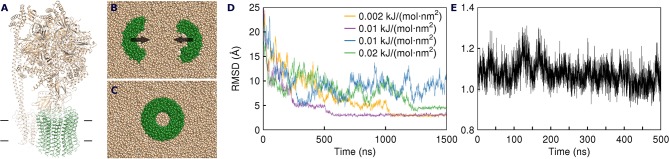
Assembly of the spinach ATP synthase rotor ring protein-lipid complex. **(A)** Structure of the spinach ATP synthase (Hahn et al., 2018). The rotor ring is highlighted in green and the approximate membrane boundaries are shown with black lines. **(B)** The rotor ring assembly approach. c_7_ oligomers are separated by 5 nm and pulled toward the experimentally determined positions using harmonic restraints. **(C)** Assembled c_14_ ring. **(D)** Convergence of the c_7_ oligomers during pulling simulations. Yellow and magenta traces converge to 3 Å; the green simulation is trapped in a high-RMSD conformation (4.6 Å); the blue simulation doesn't converge in 1,500 ns. **(E)** RMSD of C_α_ positions in unrestrained atomistic simulation of the assembled c-ring with 17 lipids inside relative to the starting experimental structure (PDB ID 6FKF).

Rotor ATPases are large multisubunit membrane protein complexes found in all kingdoms of life that convert the energy stored in transmembrane potential into the energy of a covalent bond in adenosine triphosphate (ATP), or vice versa (Walker, 2013; Junge and Nelson, 2015). Generally, ATPases consist of two large parts, one of which is soluble and another one is embedded in the membrane. The membrane part includes a so-called c-ring—a symmetric homo- or heterooligomer of subunits c, which is rotating in the membrane during the protein operation (Figure 1A). Currently, c-rings consisting of 8 to 17 protomers are known (Kühlbrandt and Davies, 2016; Schulz et al., 2017). The number of c subunits defines the energetics of ATP synthase because it corresponds to the number of ions (H^+^ or Na^+^) transported per a full 360° rotation, which invariably results in synthesis or hydrolysis of 3 ATP molecules. Most of the subunits c consist of two transmembrane helices with the connecting loop directed toward the soluble part of ATP synthase, whereas others have four transmembrane helices, which presumably results from gene duplication. Some of the c-rings can self-assemble (Arechaga et al., 2002) whereas others require accessory subunits such as UncI (Ozaki et al., 2008). The inside pore of the c-rings in some cases is plugged by phospholipids (Meier et al., 2001; Oberfeld et al., 2006).

Highly detailed atomistic structures of isolated c-rings and whole ATP synthases have recently became available (Kühlbrandt and Davies, 2016; Guo and Rubinstein, 2018; Murphy et al., 2019). High resolution crystallographic structures reveal both the overall fold as well as the geometry of the active site comprising a carboxylate amino acid, which is de- and re-protonated during the protein operation. Structures obtained using single particle cryo-electron microscopy (EM) currently have lesser resolution, but are better at revealing the overall complex structure, often in several conformations. Interestingly, in some recent EM structures of yeast V-ATPase, additional transmembrane (TM) helices have been observed inside the c-ring (Mazhab-Jafari et al., 2016; Roh et al., 2018). While the protein components of ATP synthases are generally well resolved in the structures, the densities for the surrounding lipid molecules, including those presumably trapped inside the c-ring, are of lesser quality. In the best available structure, that of *Enterococcus hirae* V-ATPase K_10_ ring, electron densities of 20 diacylglycerol/phosphatidylglycerol molecules are resolved in the immediate vicinity of the protein inside the ring, but not in its center or outside the ring (Murata et al., 2005). Most probably this is due to the great flexibility and dynamic nature of lipids (Buslaev et al., 2016) and lack of specific lipid-protein interactions, or due to detergent extraction used for sample preparation. Consequently, the identities of lipids, and their exact positions, are not well resolved in the available experimental density maps, and corresponding moieties are not modeled in experimental structures.

The lipids in and around c-rings were simulated in several previous studies, for example (Aksimentiev et al., 2004; Sengupta et al., 2009; Krah et al., 2010; Pogoryelov et al., 2010). In the smaller c_10_ rings, usually, four lipids were modeled inside (Aksimentiev et al., 2004; Sengupta et al., 2009; Zhou et al., 2017a). Larger c_14_ and c_15_ rings were simulated with 12 and 15 lipids in the central plug, respectively (Krah et al., 2010). Finally, an even larger K_10_ ring (40 TM helices) should contain even more lipids (Murata et al., 2005; Leone et al., 2015). Molecular dynamics (MD) simulations have also been used to probe the specific interactions of cardiolipin with the outer surface of metazoan c-rings (Duncan et al., 2016).

Recently, a Cryo-EM structure of spinach chloroplast ATP synthase with recognizable densities in the membrane region has become available (Hahn et al., 2018), providing an exciting opportunity to compare the modeled lipid positions with the experimental data. Neither this structure, nor previously determined crystallographic structures of similar chloroplast c_14_ rings (Vollmar et al., 2009; Saroussi et al., 2012; Balakrishna et al., 2014), contain lipid molecules. Here, we use an approach inspired by the natural assembly process to model the lipids inside the c-ring. It builds upon the existing approaches for insertion of preformed oligomers (Kandt et al., 2007; Lindahl and Sansom, 2008; Wolf et al., 2010; Biggin and Bond, 2015; Javanainen and Martinez-Seara, 2016), and uses a biasing force to assemble the whole ring, essentially by incorporating experimental restraints into the simulation. Using the approach, we observed trapping of up to 26 palmitoyl-oleoyl-phosphatidylcholine (POPC) lipids inside the c-ring, and positions of these lipids correspond well to the experimentally determined density maps. Then, we compare simulations with different numbers of lipids inside the c-ring to experimental maps and find the best fitting ones.

## Results and Discussion

Our approach to assembly of the c-ring protein-lipid complex is inspired by the natural assembly process. In nature, probably, subunits c are synthesized by ribosome independently and inserted into the membrane one-by-one. After that, the protomers assemble into oligomers, sometimes observed in atomic force microscopy images (Müller et al., 2001; Pogoryelov et al., 2005), and eventually form complete rings. Some of the c-rings can assemble in liposomes *in vitro* while others require presence of accessory proteins (Rühle and Leister, 2015). On the contrary, the existing computational methods for insertion of membrane proteins into membranes involve quite unnatural assembly steps: most of the approaches artificially insert fully assembled proteins and protein complexes into pre-equilibrated membrane patches, whereas others rely on the self-assembly of the membrane around the membrane proteins or their complexes (Kandt et al., 2007; Lindahl and Sansom, 2008; Wolf et al., 2010; Biggin and Bond, 2015; Javanainen and Martinez-Seara, 2016). In most cases, these unnatural approaches work very well, but in special cases, where some of the lipids are encapsulated within the membrane protein or protein complex, special care may be required.

Since the c subunits are relatively simple hairpin-shaped proteins, and there are no lipid molecules trapped in the c:c interfaces, we skipped the initial oligomerization steps in our simulations and started directly from partially assembled rings. Partially assembled rings have been simulated before, when the true stoichiometry of the ring was not known (Schlegel et al., 2012) or in a permeability study (Zhou et al., 2017b). Spinach c-ring contains 14 protomers, which we separated into two heptamers for simplicity of the assembly protocol. While it is likely that in nature the assembly proceeds via oligomers of different stoichiometry, we believe that the resulting arrangements of encapsulated lipids should be similar for different assembly pathways. The c_7_ heptamers, separated by 5 nm, were inserted into the membranes consisting of the commonly used model lipid POPC and were pulled toward the experimental positions by a biasing potential realized as a harmonic restraint to the experimental structure (Figures 1B,C). While theoretically the halves could assemble into a complete rotor without any biasing force, in practice this might require a large amount of computational time. Using a force too weak might also result in slow assembly. On the contrary, applying a force too strong could result in unnatural deformation of the bilayer and trapping of a system in unnatural conformation. Therefore, we tested several harmonic biasing potentials with spring constants of 0.002, 0.005, 0.01, and 0.02 kJ/(mol·nm^2^) and followed root-mean-square deviation (RMSD) of atomic positions relative to the reference experimental structure (Hahn et al., 2018). Using a coarse grained (CG) force field allowed for much faster assembly simulations, due to both computational efficiency and faster dynamics in CG simulations (Marrink and Tieleman, 2013; Buslaev and Gushchin, 2017). In many of the simulations, the structures converged into an assembled ring in <1.5 μs. The numbers of lipids trapped at the loop side and the N- and C-termini (NC)-side were consistent in different simulation runs: 9 to 11 and 13 to 15, respectively (Table 1). Using stronger restraints often resulted in trapping of the complex in a high-RMSD conformation (~4.6 Å), whereas using weaker restraints resulted in most cases in absence of convergence (Figure 1D and Table 1).

**Table 1:** Details of the assembly simulations.

**Lipid**	**Restraint coefficient, kJ/(mol·nm^**2**^)**	**# assembled (not assembled)**	**Percent assembled**	**Convergence time, μs**	**RMSD, Å**	**# of lipids, loop side**	**# of lipids, NC side**	**Total # of lipids**
POPC	0.002	1 (4)	20	1.1	3.0	10	13	23
POPC	0.005	0 (8)	0	n.d.	n.d.	n.d.	n.d.	n.d.
POPC	0.01	3 (6)	33	0.7, 1.0, 4.0	3.1, 3.1, 4.7	9, 10, 11	14, 14, 15	23, 24, 26
POPC	0.02	4 (4)	50	0.9, 0.8, 0.5, 1.4	4.6, 4.5, 3.0, 4.5	10, 10, 10, 10	15, 14, 14, 13	25, 24, 24, 23
DOPC	0.01	5 (8)	38	0.9, 1.4, 1.2, 1.5, 0.6	4.7, 4.6, 4.5, 4.6, 4.5	10, 9, 9, 10, 9	15, 14, 13, 15, 13	25, 23, 22, 24, 22
DPPC	0.01	5 (10)	33	1.0, 1.4, 2.2, 0.8, 0.6	4.5, 3.0, 4.5, 3.0, 3.4	11, 10, 10, 10, 10	14, 14, 15, 14, 14	25, 24, 25, 24, 24

*Convergence times, RMSD and numbers of lipids are indicated only for the trajectories where complete assembly has been observed*.

**Figure 2:**
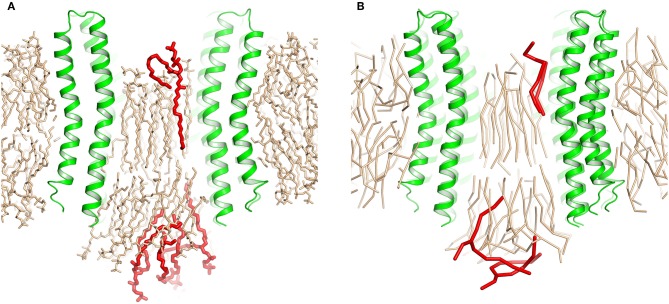
Extrusion of excess lipids from the c-ring. Position of the lipids that are pushed out (red) is shown relative to the position of other lipids (beige). **(A)** Atomistic simulation with 25 lipids. **(B)** Coarse grained simulation with 23 lipids.

After the heptamers have approached each other, we followed the initial weak restraint simulation step by a series of three consecutive 200 ps simulations with strong restraint spring constants of 10, 100, and 1,000 kJ/(mol·nm^2^), after which the RMSD relative to the experimental structure dropped below 0.8 Å. After that, the CG positions of plug lipids were converted into all atom (AA) representation using *backward* (Wassenaar et al., 2014), and the CG protein model was replaced by the experimental atomic structure. The resulting complex was solvated by water molecules and simulated without restrains. First, we conducted an AA simulation with an average amount of plug lipids (25). The c-ring remained stable but some lipids from each side of the ring were pushed out of the c-ring core (Figure 2A). Therefore, we have attempted a second simulation, starting from the converged structure with 23 plug lipids. During the strong restraint preparatory CG simulation, three lipids were also pushed out of the c-ring (Figure 2B).

**Figure 3:**
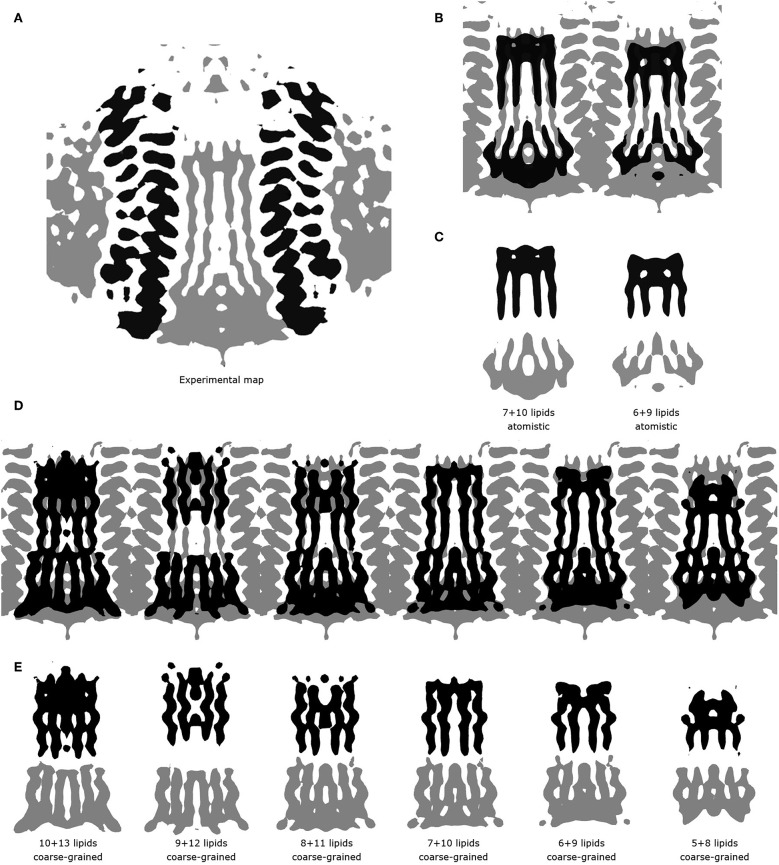
Comparison of experimental and simulated lipid densities. In all panels, the protein positions are aligned. Experimental map is shown at the level of 0.2 a.u., CG and atomistic simulated maps are shown at the level of 1.3 and 7 a.u., respectively. **(A)** 14-fold averaged Cryo-EM map. Densities corresponding to subunits c are shown in black, other densities are shown in gray. **(B)** Comparison of the results of atomistic simulations with the experimental map. Experimental map is shown in gray, simulated densities are shown in black. **(C)** Densities corresponding to lipids at the loop side (black) and to lipids at the NC-side (gray). **(D)** Comparison of the results of coarse grained simulations with the experimental map. Experimental map is shown in gray, simulated densities are shown in black. **(E)** Densities corresponding to lipids at the loop side (black) and to lipids at the NC-side (gray).

These short simulations suggested that our assembly simulations overestimated the number of lipids inside the spinach chloroplast c-ring, and prompted us to conduct a quantitative comparison of the lipid positions obtained in simulations to experiment. The original structure (Hahn et al., 2018) is not symmetrical due to presence of ATP synthase subunits other than the c-ring. Yet, the ring itself in the experimental structure is symmetric, and a free c-ring in a lipid membrane is expected to be symmetric as well. Thus, for analysis, we averaged the experimental Cryo-EM map according to the 14-fold symmetry of the ring (Figure 3). We have conducted a number of 1 μs long CG simulations of c-ring assemblies where lipids were removed from each side one-by-one, while the protein particles were restrained to the experimental positions. The densities obtained in simulations were averaged over the trajectory length and also over the 14-fold symmetry of the ring. The best fit is observed visually for the system with 6 or 7 lipids at the loop side and 9 or 10 lipids at the NC side (Figure 3D). Real space correlation coefficients (RSCC), used routinely to compare EM maps to each other and to evaluate model-density fits (Afonine et al., 2018), highlight the 6:9 system as the best fitting one with the RSCC value of ~0.75 (Table 2). The molecular entities with RSCC values above 0.7 are generally recognized as consistent with corresponding maps (Neumann et al., 2018). Overall, the 6:9 system reproduces the densities in the acyl chain region very well, but clearly lacks needed density in the head group region.

**Table 2:** Real space correlation coefficients (RSCC) for simulations with different numbers of lipids.

	**System**							
Region	7+10 lipids atomistic	6+9 lipids atomistic	10+13 lipids CG	9+12 lipids CG	8+11 lipids CG	7+10 lipids CG	6+9 lipids CG	5+8 lipids CG
Overall	0.78	0.78	0.60	0.58	0.62	0.71	0.75	0.71
Loop side	0.7	0.69	0.40	0.36	0.40	0.59	0.66	0.59
NC side	0.87	0.91	0.77	0.79	0.80	0.81	0.83	0.83

After the CG simulations, we have also conducted short atomistic simulations of 6:9 and 7:10 systems. Both revealed similar correlation with the experimental map (Table 2), with the 6:9 system severely lacking densities in the head group region, and the 7:10 system having better correspondence there (Figure 3B). The reason for the discrepancy in the head group region might be that we used the model lipid POPC in our simulations, whereas the natural thylakoid membranes contain a complex mixture of phosphatidylglycerol, monogalactosyldiacylglycerol, digalactosyldiacylglycerol and sulfoquinovosyldiacylglycerol lipids (Webb and Green, 1991). Phosphate and choline moieties of POPC have joint molecular weight of about 200 Da, whereas one galactose moiety weighs around 180 Da, two galactoses weigh ~360 Da, and sulfoquinovose weighs ~250 Da. Therefore, native lipids have head groups with higher molecular mass and bigger volume, and this might account for the additional density observed in the lipid head group region of experimentally determined density maps.

**Figure 4:**
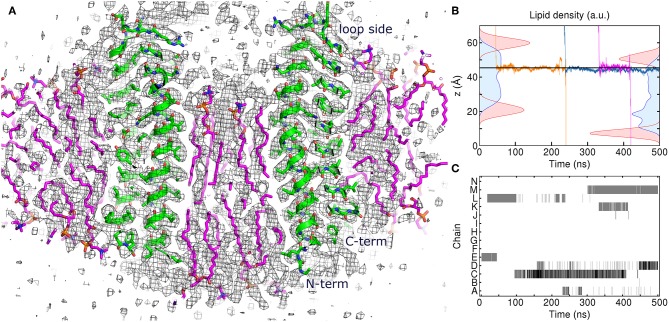
Atomistic simulation of the spinach chloroplast ATP synthase rotor ring. **(A)** Comparison of the assembled model with 17 lipids inside the c-ring at 250 ns with the experimental Cryo-EM map EMD_4273 (Hahn et al., 2018) contoured at the level of 3σ (black). Excellent correspondence between positions of protein atoms (green) and experimental map is observed. The lipids (magenta) fit very well into the densities in the membrane region. **(B)** Density distributions of outer (left) and inner (right) POPC phosphate (red) and diacylglycerol (blue) moieties overlaid with three exemplary trajectories of permeating water molecules. Average position of Glu61 centers of mass is shown using the solid line. **(C)** Contact maps between water molecules and Glu61 side chains. Presence of water molecules whose oxygen atoms are within 4 Å of Glu61 O_ε1_ and O_ε2_ atoms is indicated using gray (1 molecule) and black (2 molecules).

To gain insight into atomistic details and to probe the system stability, we have simulated the 7:10 system without any applied restraints for 500 ns. Overall, the system was stable with the protein C_α_ RMSD of ~1.1 Å (Figure 1E) and the lipids remaining close to the original position. We observe a reasonable correspondence of modeled lipid positions to the experimental densities (Figure 4A). Interestingly, the lipids encapsulated within the c-ring are displaced relative to the surrounding bilayer (Figure 4B), as it was observed before in experimental and modeled structures of other c-rings (Murata et al., 2005; Krah et al., 2010). At the loop side of the c subunit, the lipids are straightened and immobile. At the NC side, the lipids are more disordered and dynamic, forming a conical shape. Oleoyl chains of three POPC molecules at the NC side are bent so that some acyl chains are oriented along the membrane plane and perpendicular to other palmitoyl and oleoyl moieties. Finally, while no water molecules were initially placed within the membrane, we observed diffusion of several molecules into the membrane toward the active site glutamate (Figures 4B,C).

**Figure 5:**
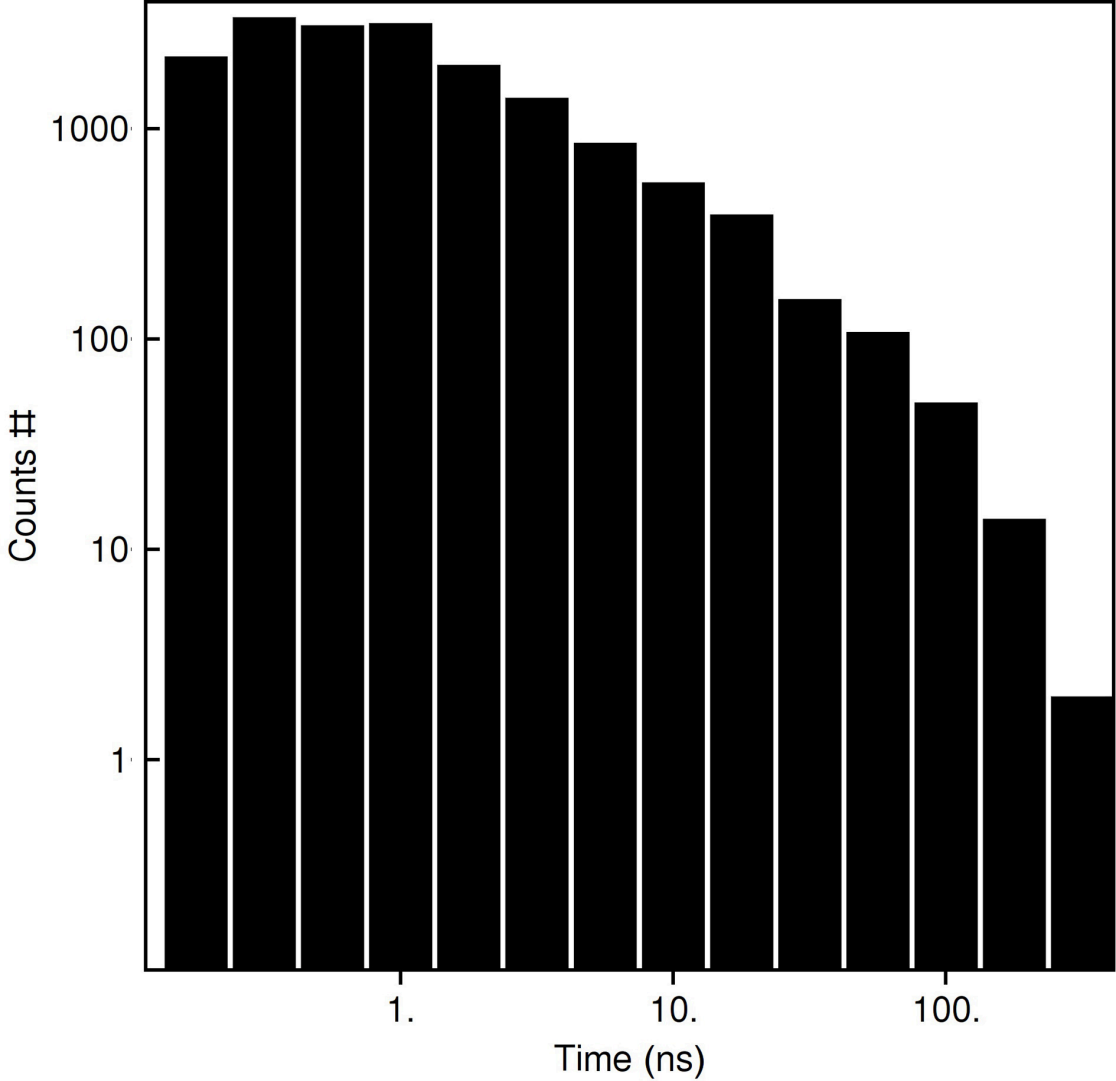
Residence times of POPC lipids in the c_7_ inner volume defined by the outermost protomers of the half-rings.

**Figure 6:**
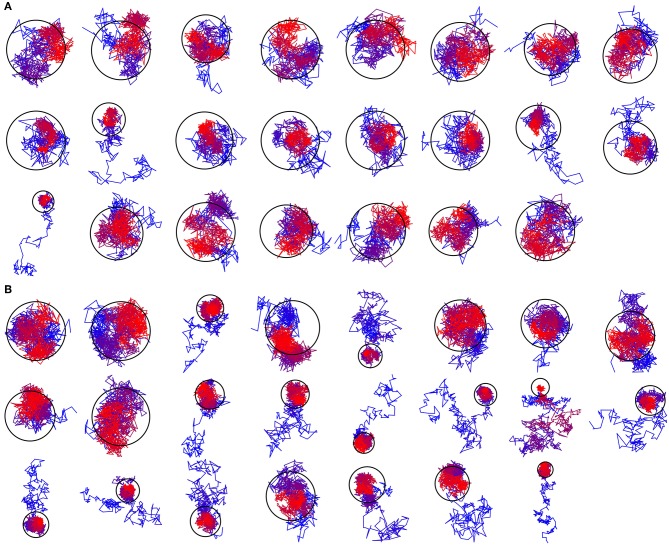
Exemplary trajectories of lipids that are eventually encapsulated inside the c-ring. The color changes gradually from blue for the starting position to red for the final position. The ring represents the assembled c-ring. **(A)** Trajectory assembled using the biasing potential coefficient of 0.01 kJ/(mol·nm^2^). Most of the encapsulated lipids are initially positioned in between the c_7_ half-rings. **(B)** Trajectory assembled using the biasing potential coefficient of 0.002 kJ/(mol·nm^2^). Most of the encapsulated lipids are initially positioned outside of the starting positions of c_7_ half-rings.

Based on the reported simulations and comparison of density distributions in the acyl chain region to the experimental data (Figure 3 and Table 2), we conclude that the experimental maps are best described by systems with 15 to 17 lipid molecules. These numbers are in contrast with 23 to 26 lipids observed in biasing potential-driven assembly simulations. Is it possible that trapping of the excessive number of lipids is a consequence of using a biasing potential? Our analyses indicate that this likely might not the case. First, most of the assemblies with the biasing potential coefficient below 0.01 kJ/(mol·nm^2^) do not converge in reasonable time (Table 1), and thus the biasing potentials are not too excessive and affect the system moderately. Second, some lipids are known to interact tightly with corresponding membrane proteins, and such interaction might prevent reaching equilibrium number of lipids inside the c-ring in accelerated assembly simulation. However, analysis of the residence times of POPC lipids in the inner volume of c_7_ half-rings separated by 5 nm reveals that lipids in most cases do not stay within the cavity for longer than ~100 ns (Figure 5). Given that the typical assembly takes 0.5–1.5 μs, the lipids have an opportunity to leave the cavity during the assembly simulation. Third, lipids are known to co-diffuse with membrane proteins (Niemelä et al., 2010), and thus might be dragged by the half-rings pulled by the biasing potential. In assembly simulations with biasing potential coefficients of 0.01 kJ/(mol·nm^2^) most of the lipids that are eventually trapped inside the c-ring are initially positioned in between the c_7_ half-rings (Figure 6A). On the contrary, in the 0.002 kJ/(mol·nm^2^) assembly simulation, the lipids are evidently free to diffuse inside and outside the space between the approaching half-rings, and most of the lipids that are eventually encapsulated are initially positioned outside of the starting positions of c_7_ half-rings (Figure 6B), yet still 23 lipids are trapped. Finally, assembly simulations with dioleoyl-phosphatidylcholine (DOPC), a lipid with two unsaturated, and strongly disordered, oleoyl chains, and dipalmitoyl-phosphatidylcholine (DPPC), a lipid with two saturated, and ordered, acyl chains result in very similar numbers of trapped lipids (Table 1). To sum up, it appears that the effects arising due to using a biasing potential, if they are present, are moderate and do not explain the difference between the numbers of 23–26 lipids trapped in the assembly simulations and 15–17 lipids observed to fit best the experimental maps. Therefore, it is tempting to speculate that during the natural assembly process the c-ring might initially include a larger number of lipids, and then consequently some lipids are extruded, as depicted in Figure 2, and either can diffuse away themselves or might be carried away by some soluble transport proteins.

Preliminary data shows that the presented approach works well also for larger K_10_ (40 TM helices) and smaller c_13_ (26 TM helices) rings (data not shown). We believe that it can be used for assembly of other protein complexes with encapsulated lipids such as the multidrug exporter AcrB (Qiu et al., 2018) or oligomeric light-driven pumps, microbial rhodopsins (Gushchin and Gordeliy, 2018; Bratanov et al., 2019; Kovalev et al., 2019) as well. As lipids are often critical for correct folding and operation of membrane proteins (Cournia et al., 2015; Hedger and Sansom, 2016; Gupta et al., 2017), reproducing their correct positions in simulations is of utmost importance.

## Methods

### Preparation of Starting Models

Starting coordinates for the spinach c-ring were taken from PDB ID 6FKF. The symmetry axis of the ring was oriented along the z axis. For assembly simulations, half-ring oligomers were separated by 5 nm along the x axis. Atomic structures were converted into Martini representation using *martinize.py* (http://cgmartini.nl/index.php/tools2/proteins-and-bilayers/204-martinize). CG lipids, ions and water particles were added using *insane* (Wassenaar et al., 2015). The resulting CG systems contained 1308 POPC molecules and 8, 15 and 25776 Na^+^, Cl^−^ and water particles, respectively, in a rectangular unit cell with dimensions of ~24 × 18 × 12 nm^3^. For atomistic simulations, the systems were reassembled using the internal lipid positions taken from CG assembly simulations (Wassenaar et al., 2014), the experimental c-ring structure, and the external lipid positions generated using CHARMM-GUI (Jo et al., 2007). The resulting AA system contained 17 lipids inside the c-ring, 242 lipids outside of the c-ring, and 7 Cl^−^ and 28348 water molecules, in a hexagonal unit cell with dimensions of ~11.5 × 11.5 × 13 nm^3^. All simulations were performed using periodic boundary conditions. pKa values of titratable amino acids were determined using PROPKA3 (Olsson et al., 2011). Active site Glu61 were protonated. Glu37 of even chains were protonated because of borderline pKa value of ~6.8. Other titratable amino acids and N- and C- termini were charged. All simulations were performed using GROMACS 5.1 (Abraham et al., 2015).

### General Details of the Simulations

CG and AA simulations were conducted using leapfrog integrator with time steps of 20 and 2 fs, at a reference temperature of 350 and 303 K, respectively, and at a reference pressure of 1 bar. Protein, lipid and water molecules were coupled to the temperature bath separately. Temperature was coupled using velocity rescale (Bussi et al., 2007) and Nosé-Hoover (Nosé, 1984) thermostats with coupling constant of 1 ps^−1^, respectively. Pressure was coupled with semiisotropic Parrinello-Rahman barostat (Parrinello and Rahman, 1981) with relaxation time of 12 or 5 ps, respectively.

### Details of CG Simulations

CG simulations were conducted using Martini 2.2 (Marrink and Tieleman, 2013) and ELNEDYN (Periole et al., 2009) force fields. The center of mass of the reference structure was scaled with the scaling matrix of the pressure coupling. The non-bonded pair list was updated every 10 steps with cutoff of 1.1 nm. Potentials shifted to zero at the cutoff of 1.1 nm and a reaction-field potential with ε_rf_ = ∞ were used for treatment of the van der Waals and electrostatics interaction as recommended (de Jong et al., 2016).

### Details of AA Simulations

AA simulations were conducted using CHARMM36 (Klauda et al., 2010; Best et al., 2012) force field. The covalent bonds to hydrogen were constrained using SHAKE algorithm (Ryckaert et al., 1977). The non-bonded pair list was updated every 20 steps with cutoff of 1.2 nm. Force-based switching function with the switching range of 1.0–1.2 nm and particle mesh Ewald (PME) method with 0.12 nm Fourier grid spacing and 1.2 nm cutoff were used for treatment of the van der Waals and electrostatics interactions.

### Details of RSCC Calculations

RSCC values have been calculated using Chimera (Pettersen et al., 2004). The experimental map sampling density is 1.053 Å in all directions, as used in the original report (Hahn et al., 2018). The simulated maps were obtained using the *volmap* utility in VMD (Humphrey et al., 1996) with the sampling density of 1 Å. The symmetry axes were determined using Chimera, and the maps were averaged with their symmetry-related images rotated by increments of 360°/14≈25.7°. The map regions where the density of the simulated maps was above 0.8 (the volume inside the c-ring) have been used for RSCC calculation. For reference, the maps shown in Figures 3D,E are drawn at the level of 1.3.

## Data Availability Statement

The datasets generated for this study are available on request to the corresponding author.

## Author Contributions

ON and PB conducted the molecular dynamics simulations, analyzed the results and helped with manuscript preparation. IG designed and supervised the project, analyzed the results, and prepared the manuscript.

### Conflict of Interest

The authors declare that the research was conducted in the absence of any commercial or financial relationships that could be construed as a potential conflict of interest.

## Abbreviations

ATP: adenosine triphosphate
POPC: palmitoyl-oleoyl-phosphatidylcholine
DOPC: dioleoyl-phosphatidylcholine
DPPC: dipalmitoyl-phosphatidylcholine
EM: electron microscopy
MD: molecular dynamics
TM: transmembrane
RMSD: root-mean-square deviation
NC: N- and C-termini
AA: all atom
CG: coarse grained
RSCC: real space correlation coefficient.

## References

[B1] Abraham M. J. Murtola T. Schulz R. Páll S. Smith J. C. Hess B. (2015). GROMACS: high performance molecular simulations through multi-level parallelism from laptops to supercomputers. SoftwareX 1–2, 19–25. 10.1016/j.softx.2015.06.001

[B2] Afonine P. V. Klaholz B. P. Moriarty N. W. Poon B. K. Sobolev O. V. Terwilliger T. C.. (2018). New tools for the analysis and validation of cryo-EM maps and atomic models. Acta Crystallogr D Struct. Biol. 74, 814–840. 10.1107/S2059798318009324 30198894 PMC6130467

[B3] Aksimentiev A. Balabin I. A. Fillingame R. H. Schulten K. (2004). Insights into the molecular mechanism of rotation in the Fo sector of ATP synthase. Biophys. J. 86, 1332–1344. 10.1016/S0006-3495(04)74205-8 14990464 PMC1303972

[B4] Arechaga I. Butler P. J. G. Walker J. E. (2002). Self-assembly of ATP synthase subunit c rings. FEBS Lett. 515, 189–193. 10.1016/S0014-5793(02)02447-X 11943219

[B5] Balakrishna A. M. Seelert H. Marx S.-H. Dencher N. A. Grüber G. (2014). Crystallographic structure of the turbine C-ring from spinach chloroplast F-ATP synthase. Biosci. Rep. 34:e00102. 10.1042/BSR20130114 27919036 PMC3971453

[B6] Best R. B. Zhu X. Shim J. Lopes P. E. M. Mittal J. Feig M. (2012). Optimization of the additive CHARMM all-atom protein force field targeting improved sampling of the backbone ϕ, ψ and Side-Chain χ1 and χ2 dihedral angles. J. Chem. Theory Comput. 8, 3257–3273. 10.1021/ct300400x 23341755 PMC3549273

[B7] Biggin P. C. Bond P. J. (2015). Molecular dynamics simulations of membrane proteins. Methods Mol. Biol. 1215, 91–108. 10.1007/978-1-4939-1465-4_5 25330960

[B8] Bratanov D. Kovalev K. Machtens J.-P. Astashkin R. Chizhov I. Soloviov D.. (2019). Unique structure and function of viral rhodopsins. Nat. Commun. 10:4939. 10.1038/s41467-019-12718-0 31666521 PMC6821725

[B9] Buslaev P. Gordeliy V. Grudinin S. Gushchin I. (2016). Principal component analysis of lipid molecule conformational changes in molecular dynamics simulations. J. Chem. Theory Comput. 12, 1019–1028. 10.1021/acs.jctc.5b01106 26765212

[B10] Buslaev P. Gushchin I. (2017). Effects of coarse graining and saturation of hydrocarbon chains on structure and dynamics of simulated lipid molecules. Sci. Rep. 7:11476. 10.1038/s41598-017-11761-5 28904383 PMC5597592

[B11] Bussi G. Donadio D. Parrinello M. (2007). Canonical sampling through velocity rescaling. J. Chem. Phys. 126:014101. 10.1063/1.2408420 17212484

[B12] Cournia Z. Allen T. W. Andricioaei I. Antonny B. Baum D. Brannigan G.. (2015). Membrane protein structure, function, and dynamics: a perspective from experiments and theory. J. Membr. Biol. 248, 611–640. 10.1007/s00232-015-9802-0 26063070 PMC4515176

[B13] de Jong D. H. Baoukina S. Ingólfsson H. I. Marrink S. J. (2016). Martini straight: boosting performance using a shorter cutoff and GPUs. Comput. Phys. Commun. 199, 1–7. 10.1016/j.cpc.2015.09.014

[B14] Duncan A. L. Robinson A. J. Walker J. E. (2016). Cardiolipin binds selectively but transiently to conserved lysine residues in the rotor of metazoan ATP synthases. PNAS 113, 8687–8692. 10.1073/pnas.1608396113 27382158 PMC4978264

[B15] Guo H. Rubinstein J. L. (2018). Cryo-EM of ATP synthases. Curr. Opin. Struct. Biol. 52, 71–79. 10.1016/j.sbi.2018.08.005 30240940

[B16] Gupta K. Donlan J. A. C. Hopper J. T. S. Uzdavinys P. Landreh M. Struwe W. B.. (2017). The role of interfacial lipids in stabilizing membrane protein oligomers. Nature 541, 421–424. 10.1038/nature20820 28077870 PMC5501331

[B17] Gushchin I. Gordeliy V. (2018). Microbial rhodopsins. Subcell. Biochem. 87, 19–56. 10.1007/978-981-10-7757-9_2 29464556

[B18] Hahn A. Vonck J. Mills D. J. Meier T. Kühlbrandt W. (2018). Structure, mechanism, and regulation of the chloroplast ATP synthase. Science 360:eaat4318. 10.1126/science.aat4318 29748256 PMC7116070

[B19] Hedger G. Sansom M. S. P. (2016). Lipid interaction sites on channels, transporters and receptors: recent insights from molecular dynamics simulations. Biochim. et Biophys. Acta Biomembranes 1858, 2390–2400. 10.1016/j.bbamem.2016.02.037 26946244 PMC5589069

[B20] Humphrey W. Dalke A. Schulten K. (1996). VMD visual molecular dynamics. J. Mol. Grap. 14, 33–38. 10.1016/0263-7855(96)00018-5 8744570

[B21] Javanainen M. Martinez-Seara H. (2016). Efficient preparation and analysis of membrane and membrane protein systems. Biochim. Biophys. Acta 1858, 2468–2482. 10.1016/j.bbamem.2016.02.036 26947184

[B22] Jo S. Kim T. Im W. (2007). Automated builder and database of protein/membrane complexes for molecular dynamics simulations. PLoS ONE 2:e880. 10.1371/journal.pone.0000880 17849009 PMC1963319

[B23] Junge W. Nelson N. (2015). ATP synthase. Annu. Rev. Biochem. 84, 631–657. 10.1146/annurev-biochem-060614-034124 25839341

[B24] Kandt C. Ash W. L. Tieleman D. P. (2007). Setting up and running molecular dynamics simulations of membrane proteins. Methods 41, 475–488. 10.1016/j.ymeth.2006.08.006 17367719

[B25] Klauda J. B. Venable R. M. Freites J. A. O'Connor J. W. Tobias D. J. Mondragon-Ramirez C.. (2010). Update of the CHARMM all-atom additive force field for lipids: validation on six lipid types. J. Phys. Chem. B 114, 7830–7843. 10.1021/jp101759q 20496934 PMC2922408

[B26] Kovalev K. Polovinkin V. Gushchin I. Alekseev A. Shevchenko V. Borshchevskiy V.. (2019). Structure and mechanisms of sodium-pumping KR2 rhodopsin. Sci. Adv. 5:eaav2671. 10.1126/sciadv.aav2671 30989112 PMC6457933

[B27] Krah A. Pogoryelov D. Meier T. Faraldo-Gómez J. D. (2010). On the structure of the proton-binding site in the F(o) rotor of chloroplast ATP synthases. J. Mol. Biol. 395, 20–27. 10.1016/j.jmb.2009.10.059 19883662

[B28] Kühlbrandt W. Davies K. M. (2016). Rotary ATPases: a new twist to an ancient machine. Trends Biochem. Sci. 41, 106–116. 10.1016/j.tibs.2015.10.006 26671611

[B29] Leone V. Pogoryelov D. Meier T. Faraldo-Gómez J. D. (2015). On the principle of ion selectivity in Na+/H+-coupled membrane proteins: experimental and theoretical studies of an ATP synthase rotor. Proc. Natl. Acad. Sci. U.S.A. 112, E1057–1066. 10.1073/pnas.1421202112 25713346 PMC4364180

[B30] Lindahl E. Sansom M. S. P. (2008). Membrane proteins: molecular dynamics simulations. Curr. Opin. Struct. Biol. 18, 425–431. 10.1016/j.sbi.2008.02.003 18406600

[B31] Marrink S. J. Tieleman D. P. (2013). Perspective on the Martini model. Chem. Soc. Rev. 42, 6801–6822. 10.1039/c3cs60093a 23708257

[B32] Mazhab-Jafari M. T. Rohou A. Schmidt C. Bueler S. A. Benlekbir S. Robinson C. V.. (2016). Atomic model for the membrane-embedded VO motor of a eukaryotic V-ATPase. Nature 539, 118–122. 10.1038/nature19828 27776355 PMC7332345

[B33] Meier T. Matthey U. Henzen F. Dimroth P. Müller D. J. (2001). The central plug in the reconstituted undecameric c cylinder of a bacterial ATP synthase consists of phospholipids. FEBS Lett. 505, 353–356 10.1016/S0014-5793(01)02837-X 11576527

[B34] Müller D. J. Dencher N. A. Meier T. Dimroth P. Suda K. Stahlberg H.. (2001). ATP synthase: constrained stoichiometry of the transmembrane rotor. FEBS Lett. 504, 219–222. 10.1016/S0014-5793(01)02708-9 11532457

[B35] Murata T. Yamato I. Kakinuma Y. Leslie A. G. W. Walker J. E. (2005). Structure of the rotor of the V-Type Na+-ATPase from Enterococcus hirae. Science 308, 654–659. 10.1126/science.1110064 15802565

[B36] Murphy B. J. Klusch N. Langer J. Mills D. J. Yildiz Ö. Kühlbrandt W. (2019). Rotary substates of mitochondrial ATP synthase reveal the basis of flexible F1-Fo coupling. Science 364:eaaw9128. 10.1126/science.aaw9128 31221832

[B37] Neumann P. Dickmanns A. Ficner R. (2018). Validating resolution revolution. Structure 26:1678 10.1016/j.str.2018.10.028 30517886

[B38] Niemelä P. S. Miettinen M. S. Monticelli L. Hammaren H. Bjelkmar P. Murtola T.. (2010). Membrane proteins diffuse as dynamic complexes with lipids. J. Am. Chem. Soc. 132, 7574–7575. 10.1021/ja101481b 20469857

[B39] Nosé S. (1984). A unified formulation of the constant temperature molecular dynamics methods. J. Chem. Phys. 81, 511–519. 10.1063/1.447334

[B40] Oberfeld B. Brunner J. Dimroth P. (2006). Phospholipids occupy the internal lumen of the c ring of the ATP synthase of *Escherichia coli*. Biochemistry 45, 1841–1851. 10.1021/bi052304+ 16460030

[B41] Olsson M. H. M. Søndergaard C. R. Rostkowski M. Jensen J. H. (2011). PROPKA3: consistent treatment of internal and surface residues in empirical pKa predictions. J. Chem. Theory Comput. 7, 525–537. 10.1021/ct100578z 26596171

[B42] Ozaki Y. Suzuki T. Kuruma Y. Ueda T. Yoshida M. (2008). UncI protein can mediate ring-assembly of c-subunits of FoF1-ATP synthase *in vitro*. Biochem. Biophys. Res. Commun. 367, 663–666. 10.1016/j.bbrc.2007.12.170 18182163

[B43] Parrinello M. Rahman A. (1981). Polymorphic transitions in single crystals: a new molecular dynamics method. J. Appl. Phys. 52, 7182–7190. 10.1063/1.328693

[B44] Periole X. Cavalli M. Marrink S.-J. Ceruso M. A. (2009). Combining an elastic network with a coarse-grained molecular force field: structure, dynamics, and intermolecular recognition. J. Chem. Theory Comput. 5, 2531–2543. 10.1021/ct9002114 26616630

[B45] Pettersen E. F. Goddard T. D. Huang C. C. Couch G. S. Greenblatt D. M. Meng E. C.. (2004). UCSF Chimera–a visualization system for exploratory research and analysis. J. Comput. Chem. 25, 1605–1612. 10.1002/jcc.20084 15264254

[B46] Pogoryelov D. Krah A. Langer J. D. Yildiz Ö. Faraldo-Gómez J. D. Meier T. (2010). Microscopic rotary mechanism of ion translocation in the F(o) complex of ATP synthases. Nat. Chem. Biol. 6, 891–899. 10.1038/nchembio.457 20972431

[B47] Pogoryelov D. Yu J. Meier T. Vonck J. Dimroth P. Muller D. J. (2005). The c15 ring of the Spirulina platensis F-ATP synthase: F1/F0 symmetry mismatch is not obligatory. EMBO Rep. 6, 1040–1044. 10.1038/sj.embor.7400517 16170308 PMC1371026

[B48] Qiu W. Fu Z. Xu G. G. Grassucci R. A. Zhang Y. Frank J.. (2018). Structure and activity of lipid bilayer within a membrane-protein transporter. PNAS 115, 12985–12990. 10.1073/pnas.1812526115 30509977 PMC6304963

[B49] Roh S.-H. Stam N. J. Hryc C. F. Couoh-Cardel S. Pintilie G. Chiu W.. (2018). The 3.5-Å CryoEM structure of nanodisc-reconstituted yeast vacuolar ATPase Vo proton channel. Mol. Cell 69, 993–1004.e3. 10.1016/j.molcel.2018.02.006 29526695 PMC5893162

[B50] Rühle T. Leister D. (2015). Assembly of F1F0-ATP synthases. Biochim. Biophys. Acta 1847, 849–860. 10.1016/j.bbabio.2015.02.005 25667968

[B51] Ryckaert J.-P. Ciccotti G. Berendsen H. J. C. (1977). Numerical integration of the cartesian equations of motion of a system with constraints: molecular dynamics of n-alkanes. J. Comput. Phys. 23, 327–341. 10.1016/0021-9991(77)90098-5

[B52] Saroussi S. Schushan M. Ben-Tal N. Junge W. Nelson N. (2012). Structure and flexibility of the C-ring in the electromotor of rotary FoF1-ATPase of pea chloroplasts. PLoS ONE 7:e43045 10.1371/journal.pone.0043045 23049735 PMC3458034

[B53] Schlegel K. Leone V. Faraldo-Gómez J. D. Müller V. (2012). Promiscuous archaeal ATP synthase concurrently coupled to Na+ and H+ translocation. Proc. Natl. Acad. Sci. U.S.A. 109, 947–952. 10.1073/pnas.1115796109 22219361 PMC3271924

[B54] Schulz S. Wilkes M. Mills D. J. Kühlbrandt W. Meier T. (2017). Molecular architecture of the N-type ATPase rotor ring from Burkholderia pseudomallei. EMBO Rep. 18, 526–535. 10.15252/embr.201643374 28283532 PMC5376962

[B55] Sengupta D. Rampioni A. Marrink S.-J. (2009). Simulations of the c-subunit of ATP-synthase reveal helix rearrangements. Mol. Memb. Biol. 26, 422–434. 10.3109/09687680903321073 19878046

[B56] Vollmar M. Schlieper D. Winn M. Büchner C. Groth G. (2009). Structure of the c14 rotor ring of the proton translocating chloroplast ATP synthase. J. Biol. Chem. 284, 18228–18235. 10.1074/jbc.M109.006916 19423706 PMC2709358

[B57] Walker J. E. (2013). The ATP synthase: the understood, the uncertain and the unknown. Biochem. Soc. Trans. 41, 1–16. 10.1042/BST20110773 23356252

[B58] Wassenaar T. A. Ingólfsson H. I. Böckmann R. A. Tieleman D. P. Marrink S. J. (2015). Computational lipidomics with insane: a versatile tool for generating custom membranes for molecular simulations. J. Chem. Theory Comput. 11, 2144–2155. 10.1021/acs.jctc.5b00209 26574417

[B59] Wassenaar T. A. Pluhackova K. Böckmann R. A. Marrink S. J. Tieleman D. P. (2014). Going backward: a flexible geometric approach to reverse transformation from coarse grained to atomistic models. J. Chem. Theory Comput. 10, 676–690. 10.1021/ct400617g 26580045

[B60] Webb M. S. Green B. R. (1991). Biochemical and biophysical properties of thylakoid acyl lipids. Biochim. et Biophys. Acta Bioenerget. 1060, 133–158. 10.1016/S0005-2728(09)91002-7

[B61] Wolf M. G. Hoefling M. Aponte-Santamaría C. Grubmüller H. Groenhof G. (2010). g_membed: Efficient insertion of a membrane protein into an equilibrated lipid bilayer with minimal perturbation. J. Comput. Chem. 31, 2169–2174. 10.1002/jcc.21507 20336801

[B62] Zhou W. Leone V. Krah A. Faraldo-Gómez J. D. (2017a). Predicted structures of the proton-bound membrane-embedded rotor rings of the *Saccharomyces cerevisiae* and *Escherichia coli* ATP synthases. J. Phys. Chem. B 121, 3297–3307. 10.1021/acs.jpcb.6b08051 27715045 PMC5593136

[B63] Zhou W. Marinelli F. Nief C. Faraldo-Gómez J. D. (2017b). Atomistic simulations indicate the c-subunit ring of the F1Fo ATP synthase is not the mitochondrial permeability transition pore. Elife 6:e23781 10.7554/eLife.23781 28186490 PMC5323039

